# Taking Root: Enduring Effect of Rhizosphere Bacterial Colonization in Mangroves

**DOI:** 10.1371/journal.pone.0014065

**Published:** 2010-11-22

**Authors:** Newton C. M. Gomes, Daniel F. R. Cleary, Fernando N. Pinto, Conceição Egas, Adelaide Almeida, Angela Cunha, Leda C. S. Mendonça-Hagler, Kornelia Smalla

**Affiliations:** 1 CESAM and Department of Biology, University of Aveiro, Aveiro, Portugal; 2 Institute of Biology, Federal University of Rio de Janeiro, Rio de Janeiro, Brazil; 3 Biocant-Biotechnology Innovation Center, Cantanhede, Portugal; 4 Center for Neurosciences and Cellular Biology, University of Coimbra, Coimbra, Portugal; 5 Institute of Microbiology, Federal University of Rio de Janeiro, Rio de Janeiro, Brazil; 6 Julius Kühn-Institute for Cultivated Crops, Braunschweig, Germany; Loyola University Medical Center, United States of America

## Abstract

**Background:**

Mangrove forests are of global ecological and economic importance, but are also one of the world's most threatened ecosystems. Here we present a case study examining the influence of the rhizosphere on the structural composition and diversity of mangrove bacterial communities and the implications for mangrove reforestation approaches using nursery-raised plants.

**Methodology/Principal Findings:**

A barcoded pyrosequencing approach was used to assess bacterial diversity in the rhizosphere of plants in a nursery setting, nursery-raised transplants and native (non-transplanted) plants in the same mangrove habitat. In addition to this, we also assessed bacterial composition in the bulk sediment in order to ascertain if the roots of mangrove plants affect sediment bacterial composition. We found that mangrove roots appear to influence bacterial abundance and composition in the rhizosphere. Due to the sheer abundance of roots in mangrove habitat, such an effect can have an important impact on the maintenance of bacterial guilds involved in nutrient cycling and other key ecosystem functions. Surprisingly, we also noted a marked impact of initial nursery conditions on the rhizosphere bacterial composition of replanted mangrove trees. This result is intriguing because mangroves are periodically inundated with seawater and represent a highly dynamic environment compared to the more controlled nursery environment.

**Conclusions/Significance:**

In as far as microbial diversity and composition influences plant growth and health, this study indicates that nursery conditions and early microbial colonization patterns of the replants are key factors that should be considered during reforestation projects. In addition to this, our results provide information on the role of the mangrove rhizosphere as a habitat for bacteria from estuarine sediments.

## Introduction

Mangrove forests are unique and diverse coastal ecosystems located in tropical and subtropical regions. These forests are both ecologically and economically important. In addition to protecting coastal areas from erosion, mangroves also diminish the impact of Tsunamis and serve as critical nurseries for juvenile fish [1], [2]. Despite the well known benefits of maintaining healthy mangroves, they are highly threatened ecosystems and at present are disappearing at a rate of 1 to 2% per year across their range [3]. Due to the growing concern that mangroves may disappear in a relatively short time frame (∼100 years) [3] and the need to reverse ongoing destruction, several international and community-based rehabilitation programs have been established across the globe [4], [5]. International organizations that support mangrove rehabilitation include the European Union, the World Bank and the World Wide Fund for Nature. In 2005, for example, the EU Commission funded a project for mangrove restoration in Sri Lanka, which resulted in more than 60,000 replanted mangrove saplings [5].

Natural regeneration is often the first strategy to be adopted for recovery of degraded mangroves. When this is hampered, restoration projects may be established that involve growing mangrove seedlings in nurseries and subsequently transplanting these to degraded areas [6]–[8]. However, reforestation approaches using nursery-raised plants often show highly variable survival rates and knowledge is lacking about the biology of the whole process. Surprisingly, despite the well-known mutual dependence between plant roots and soil microbial communities [9], [10], no studies have hitherto made an in depth analysis of how initial growth conditions and transplantation affect the microbial communities of replants. The interaction between plants and microorganisms has in fact only recently become a focal topic in restoration ecology [11]. Previous studies have demonstrated that soil microorganisms are essential for nutrient cycling, soil structure generation and decomposition and are thus key players in the regulation of plant productivity and plant community dynamics [11]. Several plant species have been, furthermore, shown to influence the microorganisms colonizing their root environment (the ‘rhizosphere effect’) [12]–[14]. In return, the microorganisms contribute to plant growth and health by nutrient solubilisation, N_2_ fixation, the production of plant hormones and the degradation of phytotoxic compounds [9], [10]. However, intertidal zones of the mangrove forests are periodically inundated and it is unknown whether roots from mangrove plants located in these extreme environments can impose a similar selective pressure on microbial communities as has been demonstrated for purely terrestrial plants.

Three key questions need to be addressed in order to ascertain whether manipulation of the microbial community in the rhizosphere can be exploited in the restoration of mangrove habitats. First of all, it is essential to investigate if mangrove plants can influence the composition of microorganisms colonizing the sediment surrounding their roots as has been observed for terrestrial plants [12]–[14]. Next, it is important to ascertain whether the initial growth conditions of nursery raised trees have a long-term effect on the microbial community of replant rhizospheres. Finally, it is necessary to evaluate if microbial rhizoengineering during initial growth conditions in the nursery can enhance plant growth and survival. Various studies have already demonstrated that plant diversity can influence such ecosystem processes as stability, productivity, nutrient dynamics and vulnerability to invasive species [15] although this remains to be shown for microbial diversity. A more diverse microbial community may, however, buffer a plant from potentially dangerous pathogens and include a diverse array of functional groups of species that facilitate plant growth.

In this study, we address the first two questions, namely if mangrove plants influence the composition of bacterial communities colonizing the sediment surrounding their roots (rhizosphere effect) and if the initial growth conditions have a significant and long-term effect on the bacterial community of replanted mangrove trees. We also compare the microbial communities of bulk sediment and the rhizosphere of native mangrove plants. In addition to comparing microbial diversity and composition among treatments, we also make an in depth analysis of the dominant bacterial populations in order to see if known beneficial microbes are enhanced in transplants and native mangrove plants compared to the bulk sediment.

## Results

**Figure 1:**
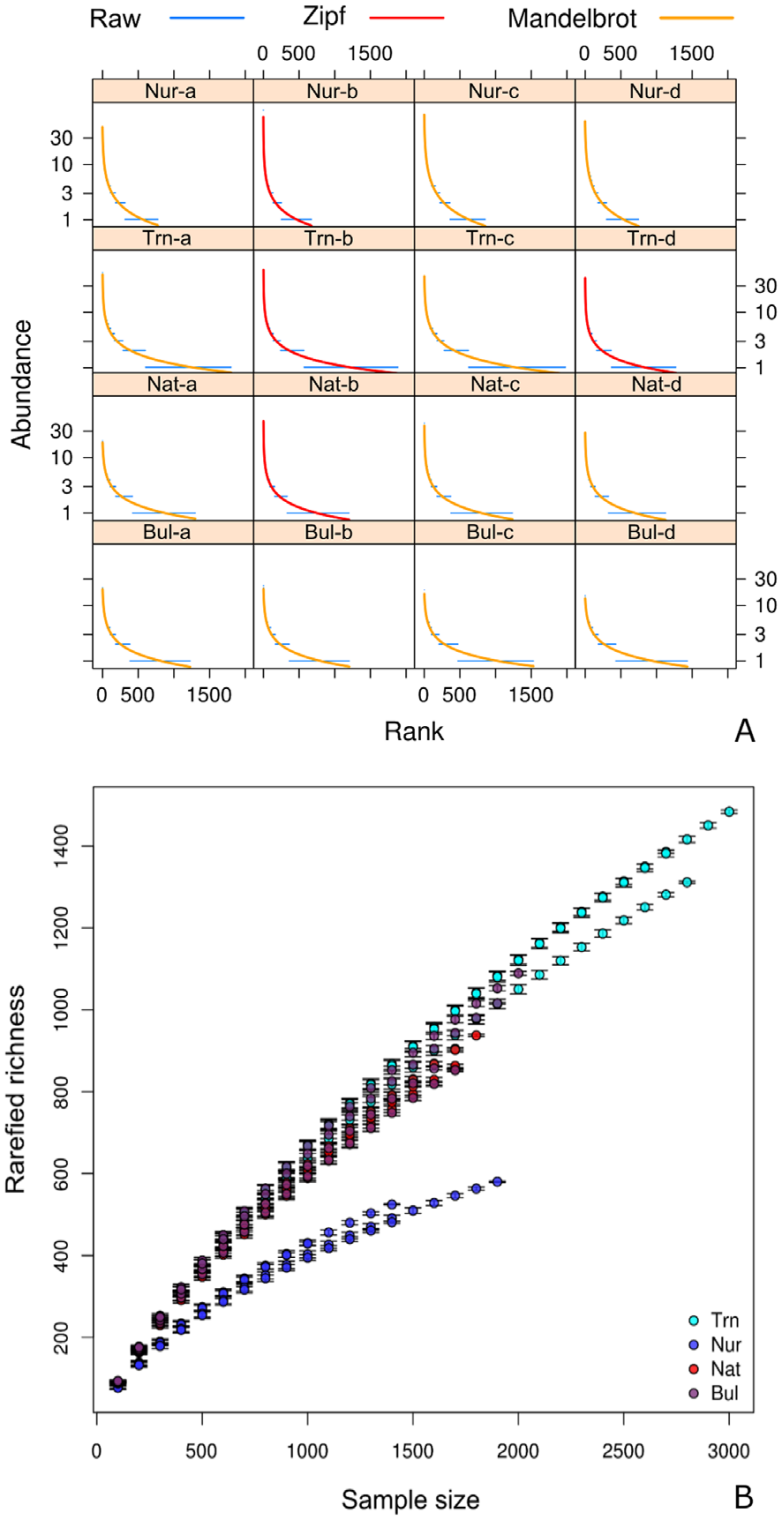
Diversity analyses of rhizosphere samples from nursery (Nur), transplanted (Trn) and native (Nat) *Rhizophora mangle* plants and from the bulk sediment (Bul). A) Dominance-diversity plots. Each panel plots logarithmic species abundance against the rank order of species for each sample. The blue horizontal lines represent observed (raw) data. The red and yellow lines represent the best fits, namely Zipf and Zipf-Mandelbrot models respectively. The best fits were obtained with the ‘radfit()’ function in the vegan library in R. B) Species rarefaction curve of each sample data set using; error bars represent a single standard deviation.

The data retrieved from the cluster analyses of the Ribosomal Database Project (RDP) pyrosequencing pipeline was used to estimate operational taxonomic units (OTU's) richness and compare composition among treatments. The dominance-diversity plots and species rarefaction curve of each sample revealed marked differences among treatments (Fig. 1A,B). Samples taken from transplanted (Trn) plants had the highest number of OTU's. Nursery (Nur) samples, in contrast, exhibited pronounced dominance of a few OTU's but contained much fewer OTU's compared to samples from other treatments. In the Bulk sediment (Bul) samples, the dominance of the most abundant OTU's was much less pronounced but there were more ‘rare’ OTU's than in the nursery samples. Rhizosphere samples from the native (Nat) saplings exhibited somewhat more dominance and fewer ‘rare’ OTU's compared to the sediment samples. The rhizosphere effect on bacterial diversity is, however, much more pronounced for transplanted samples that were raised in a ‘terrestrial’ soil matrix. In addition to the pronounced dominance, the transplanted samples also contained a very large proportion of ‘rare’ OTU's compared to samples from other treatments.

**Figure 2:**
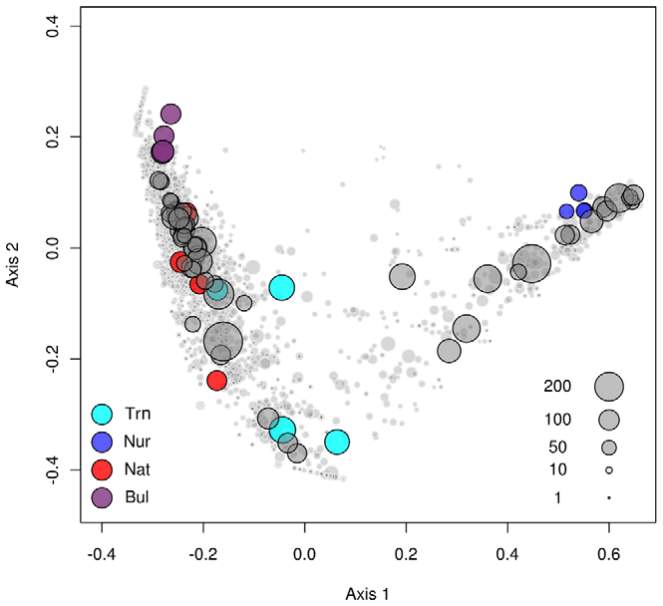
Principal coordinates (PCO) analysis of operational taxonomic unit (OTU) composition. The first two axes of a PCO ordination are shown based on a matrix of OTU composition of rhizosphere samples from nursery (Nur), transplanted (Trn) and native (Nat) *Rhizophora mangle* plants and from the bulk sediment (Bul). Grey symbols represent individual OTU's where the size of the symbol corresponds to its total abundance (see legend in plot). Coloured symbols represent sample sites where the size corresponds to OTU richness.

There was significant variation in OTU composition among treatments (Adonis analysis: F_3,15_ = 3.518, R^2^ = 0.468, P<0.001). A principal coordinates analysis (PCO), using the Hellinger distance, of OTU composition (Fig. 2) showed that the primary axis of variation was between samples obtained from the nursery and samples from the rhizosphere and bulk sediment in the mangrove sampling site. The transplanted samples, however, had the greatest similarity (of the mangrove samples) to the nursery samples, with several dominant OTU's in common. Along Axis 2, the greatest difference was between the transplanted and the bulk sediment samples; native plant samples were intermediate.

**Figure 3:**
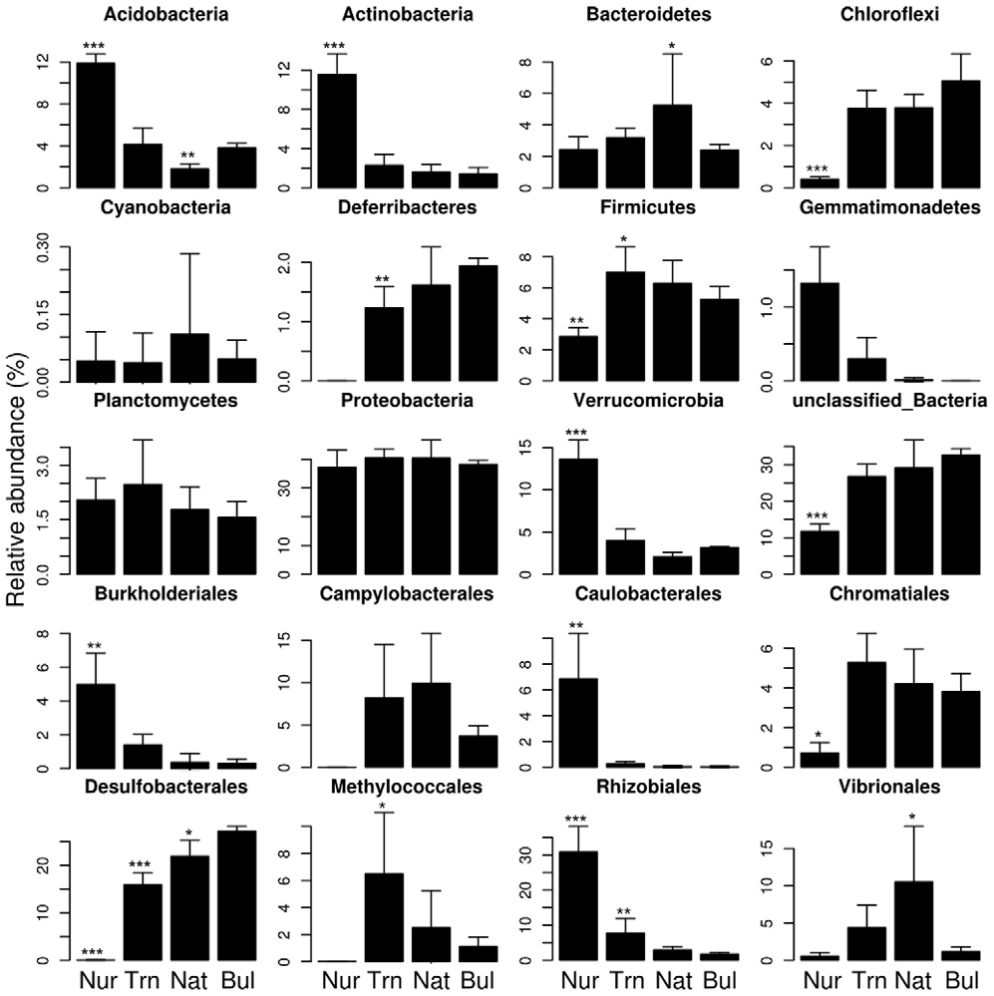
Relative abundance of the most dominant bacterial groups. Each panel plots the relative abundance of different bacterial taxa inhabiting rhizosphere samples from nursery (Nur), transplanted (Trn) and native (Nat) *Rhizophora mangle* plants and bulk sediments (Bul). All classes are shown where the relative abundance in at least one treatment exceeds 1% (first three rows). The eight most abundant classified orders of *Proteobacteria* are also shown (last two rows). Symbols above the bars represent significant deviations (*** P<0.001, ** 0.001<P<0.01, * 0.01<P<0.05) from the relative abundance in the bulk sediment using an analysis of deviance (glm with ‘quasibinomial’ family). Note that we did not control for multiple tests.

In line with the PCO, the RDP classification of the OTU's showed that the nursery samples contained the most distinct composition of the major taxonomic groups, e.g., significantly higher relative abundances of *Acidobacteria*, *Actinobacteria*, *Verrucromicrobia*, *Burkholderiales*, *Caulobacterales* and *Rhizobiales* and significantly lower relative abundances of *Chloroflexi*, *Firmicutes* and *Desulfobacterales* (Fig. 3). Nursery samples also contained fewer phyla than the mangrove samples. Interestingly, the *Bacteroidetes* were markedly more abundant in the rhizosphere of native plants than in either the nursery or bulk sediment samples. *Proteobacteria* was the most abundant phylum in all samples and comprised from 36% to 40% of total reads. The relative abundance of the most dominant orders within the *Proteobacteria* are also shown in Fig. 3. *Desulfobacterales* was the most abundant proteobacterial order detected in Trn, Nat and Bul samples, with 17, 23 and 30% of the total reads assigned to this order, respectively. *Chromatiales* was the second most abundant order, almost equally distributed among mangrove samples, with only a few representatives detected in nursery samples.

**Figure 4:**
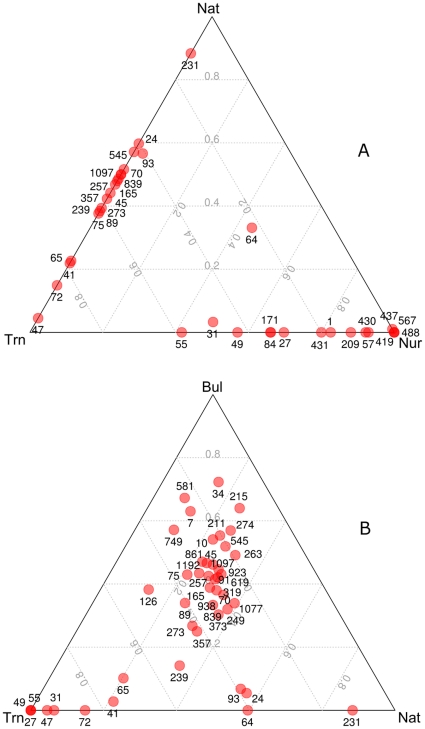
Ternary plots showing the ratio's of the most abundant operational taxonomic units (OTU's) across treatments [rhizosphere samples of nursery (Nur), transplanted (Trn), native *Rhizophora mangle* (Nat) and bulk sediment (Bul)]. Ratio's in rhizosphere (Nur, Trn and Nat) treatments (A) and mangrove (Trn, Nat and Bul) treatments (B) are shown. Numbers identify individual OTU's (see Table 1). In ternary plots each corner of the triangle represents a proportion of 100% for a given treatment with the other corners representing 0% of that treatment. As the proportion of a given treatment increases in a sample then it moves towards the corner representing that treatment.

Of the orders shown in Fig. 3, *Rhizobiales*, *Campylobacterales*, *Methylococcales* and *Vibrionales* tend to be more abundant in the rhizosphere samples than in the bulk sediment. *Rhizobiales* populations were interestingly also significantly more abundant in nursery and transplant samples than in native and bulk sediment samples. Transplant rhizospheres showed about three times more abundant OTU's assigned to *Rhizobiales* than native plants. The relative abundance of this order in nursery samples was in turn about five times higher than in transplanted samples. The OTU's assigned to *Methylococcales* were all assigned to the family *Methylococcaceae* (mainly *Methylomonas*) (using the RDP Classifier) and were at least twice as abundant in mangrove rhizosphere samples (Trn and Nat) than in bulk sediment. Curiously, in Fig. 4A (the ternary plot of dominant OTU's distributed in the rhizosphere samples) specific OTU's related to aerobic methanotrophs (41 and 47) assigned to members of the family *Methylococcaceae* (Table 1), were specifically enhanced in the transplanted plants. The abundance and diversity of *Vibrionales* was also much higher in mangrove rhizosphere samples than either nursery or bulk sediment samples. The great majority of these OTU's were assigned to the genus *Vibrio* (using the RDP Classifier).

**Table 1:** Taxonomic assignment of partial 16S rRNA gene sequences of dominant bacterial populations (operational taxonomic units ≥50 reads) and their known putative ecophysiological traits.

OTU code/number of reads	Sequence classification	Known traits
**839**/78, **545**/131, **249**/50, **7**/73, **91**/66, **64**/160, **257**/127, **45**/148, **165**/121	***Bacteria***	Unknown *Phylum*.
**923**/65	***Proteobacteria***	The *Phylum Proteobacteria* is highly diverse, widely distributed in marine environments, and is known as an important player in the process of nutrient cycling.
**171**/62, **274**/72, **1097**/104, **75**/135, **72**/94**619**/76**231**/60**437**/199**41**/108, **47**/89**1192**/53**211**/103, **319**/77, **880**/50**93**/363	***Gammaproteobacteria*** *Haliea* *Listonella* *Marinomonas* *Methylomonas* *Thiohalocapsa* *Thiohalophilus* *Vibrio*	The *Gammaproteobacteria* includes populations that are able to decompose marine organic matter. Many OTU's classified into this Class are known for their importance in nutrient cycling in marine ecosystems. However, *Haliea* strains were recently isolated from marine environments and their putative ecological functions are still unknown.The genus *Listonella* includes diazotrophic populations with some representatives previously detected in mangrove rhizospheres.*Marinomonas* species have been previously isolated from salt marsh roots and evidence suggests that members of this genus are involved in dimethylsulphoniopropionate (DMSP) catabolism in the rhizosphere of estuarine plants. The degradation of DMSP into dimethylsulfide is a key process in the transfer of sulphur from marine ecosystems to the atmosphere.The family *Methylococcaceae* includes aerobic methanotrophs such as *Methylomonas* spp. This family belongs to a group of methanotrophic bacteria, which are important in reducing the methane flux from sediment (marine and fresh water) to the atmosphere.Members of the *Thiohalocapsa* genus are purple sulfur photolithoautotrophic bacteria with halophilic growth response. The *Thiohalophilus* genus comprises sulphur-oxidizing chemolithoautotrophic bacteria which are also capable of halophilic growth.The *Vibrio* genus includes mainly aquatic bacteria of which several are free-living and obligate endosymbionts.
**419**/53**431**/90**1**/86, **49**/186**567**/98	***Alphaproteobacteria*** *Rhizobiales* *Bradyrhizobium* *Asticcacaulis*	The Alphaproteobacteria comprise several plant symbionts. In this study several OTU's assigned to this class were related to rhizobia (*Rhizobiales*); these are well known for their ability to fix atmospheric nitrogen in association with plants. *Bradyrhizobium* spp. are well known as root-nodule bacteria, which are used as plant inoculants worldwide.The cluster 567 was assigned to the genus *Asticcacaulis*, which consists of chemoorganotrophic, aerobic organisms.
**84**/50	***Betaproteobacteria*** *Herbaspirillum*	The genus *Herbaspirillum* also comprises endophytic diazotrophs.
**70**/100**34**/65, **861**/68**273**/71**89**/209**126**/68**24**/92, **581**/58, **1077**/59**938**/52	***Deltaproteobacteria*** *Desulfobacteraceae* *Desulfatibacillum* *Desulfosarcina* *Desulfobacterium* *Desulfobulbaceae* *Desulfobulbus* *Syntrophobacterales*	*Deltaproteobacteria*, have been described as a key group of sulphate-reducing bacteria (SRB) in marine sediments. The *Desulfobacteraceae* (*Desulfatibacillum*, *Desulfosarcina* and *Desulfobacterium*) and *Desulfobulbaceae* (*Desulfobulbus*) families are often detected in marine environments and are important players in the process of anoxic mineralization of organic matter.
**357**/160**65**/59, **239**/219	***Epsilonproteobacteria*** *Helicobacteraceae* *Sulfurovum*	Members of the *Helicobacteraceae* family are involved in autotrophic nitrate reduction and sulfide oxidation. The genus *Sulfurovum* (*Campylobacterales*) is an important player in the process of sulfide-oxidation and denitrification in marine environments.
**57**/102**215**/75	***Acidobacteria*** *Gp1* *Gp23*	Only a few *Acidobacteria* strains have been cultivated up to now. Therefore, the putative ecological function of this group still remains largely unknown.
**749**/56	***Actinobacteria*** *Actinomycetales*	Members of this order are best known from soils and plant rhizospheres. They are well known as efficient degraders of complex biopolymers (e.g. lignocellulose, keratin, and chitin). Only recently the actinomycetes were recognized as autochthonous marine microbiota
**55**/132	***Bacteroidetes***	The *Chitinophagaceae* family includes isolates that have been frequently detected in several environmental samples and are well known as efficient degraders of biopolymers.
	*Chitinophagaceae*	
**10**/61	***Chloroflexi***	Members of this phylum consist of facultatively aerobic, filamentous bacteria and are presumably involved in the degradation of carbohydrates and amino acids.
**263**/57, **373**/66	***Deferribacteres*** *Caldithrix*	The *Deferribacteres* class includes species involved in dissimilatory Fe reduction. The genus *Caldithrix* includes a few isolates retrieved from extreme environments which were nitrate-reducing, anaerobe chemo-organoheterotrophs capable of fermenting proteinaceous substrates.
**488**/63**27**/350, **209**/126**31**/183, **430**/99	***Verrucomicrobia*** *Opitutus* *Spartobacteria_genera_ incertae_sedis* *Subdivision3_genera_ incertae_sedis*	*Verrucomicrobia* OTU's are frequently found in culture-independent surveys of a broad range of environmental and non-environmental samples. However, there are only a few species belonging to this group which have been successfully isolated and cultivated. In general they are mesophilic carbohydrate degraders. Recently a few aerobic methanotrophs of *Verrucomicrobia* have been found.

The codes in bold refer to the OTUs' code followed by the number of sequences reads assigned to each OTU.

Sequences were assigned up to the lowest taxonomic rank with at least 50% bootstrap support.

The ternary plots of dominant OTU's (Fig. 4A,B) show a dominant bacterial population (OTU 64), which was almost equally distributed in all rhizosphere samples, but was not detected in the bulk sediment. This OTU was assigned to *Bacteria* with high confidence (94%) but could not be assigned to any known *Phylum* (Table 1). Three dominant OTU's (1, 49 and 431) found in nursery and transplanted rhizospheres, were classified as diazotrophic bacteria belonging to the order *Rhizobiales* (Table 1). Several other dominant OTU's were much more abundant or only detected in the nursery samples and seem not to be able to persist in the mangrove environment after transplantation; for example, members of the order *Caulobacterales*, which are known chemoorganotrophic aerobic organisms.

The relative abundance of dominant OTU's present in the mangrove samples (Fig. 4B) revealed several OTU's with strong associations to the mangrove rhizospheres (Trn and Nat). While the OTU's 24, 93 and 231 were more abundant in Nat rhizospheres, OTU's 27, 31, 41, 47, 49, 65, 72 and 239 were more prevalent in Trn rhizospheres. The taxonomic assignment of these OTU's and their known putative ecophysiological traits are presented in Table 1. In general, in agreement with the relative abundance analyses of the order *Campylobacterales* (Fig. 3), OTU's assigned to the genus *Sulfurovum* (65 and 239) were more abundant in the rhizosphere samples (with stronger associations to transplants) but were again rare in the bulk sediment (Fig. 4B and see Table 1). The rhizosphere of transplants also appeared to have enhanced the colonization of bacterial populations related to *Methylomonas* (OTU's 41 and 47). In contrast, OTU's assigned to the diazotrophic bacteria *Listonella* (231) and *Vibrio* (93) were mainly detected in the rhizosphere of native plants.

In this study, some dominant bacterial OTU's associated to known plant-beneficial organisms were only detected in nursery or transplanted samples. Rhizobacterial populations acquired during nursery growth were, therefore, presumably able to survive in the mangrove environment and remained abundant in the rhizosphere up to 202 days after planting (dap). Several dominant OTU's, furthermore, showed specific associations with the rhizosphere of native and transplanted plants and were assigned to microbial guilds known to influence nutrient cycling (Fig. 3, Fig. 4A,B; also see Table 1).

## Discussion

The mutual dependence between plants and microbes is a fundamental biological interaction that has been largely ignored in mangrove ecology and virtually all mangrove reforestation projects. Mangroves are unique coastal marine intertidal environments and as such are periodically inundated with seawater. Therefore the mangrove root bacterial communities have to adapt to living in a habitat which is exposed to extreme changes on a daily basis due to tidal cycles. In this study, diversity plots and PCO analysis revealed marked differences between rhizosphere (Nur, Trn and Nat) and bulk sediment communities. These results agree with the concept of the so-called ‘rhizosphere effect’, a phenomenon well described for terrestrial plants. The rhizosphere effect is typically characterized by a reduced diversity in the rhizosphere compared to the bulk sediment and increased abundance of root specialized bacterial guilds [16]. This effect in the mangrove plants is, however, much more pronounced for transplants that were raised in a ‘terrestrial’ soil matrix. In addition to the pronounced dominance, the transplants showed a larger proportion of ‘rare’ OTU's compared to bulk sediment. This is, therefore, an atypical rhizosphere effect and suggests that the unique initial growth conditions (for mangrove plants) and transplantation, as it was performed in this study, has had a marked impact on the diversity and composition of the bacterial communities of replants.

The RDP classifier analysis of all bacterial OTU's revealed several bacterial guilds colonizing mangrove samples which are known for their importance in the marine biogeochemical cycling of carbon, nitrogen and sulphur. Marine members of the *Bacteroidetes* were more abundant in the rhizosphere of native plants and are known degraders of particulate organic matter in the ocean [17]. However, their potential ecological role in mangrove rhizospheres is unknown. The *Proteobacteria* was the most abundant phylum in all samples. This group is metabolically highly diverse, widely distributed in marine environments, and is an important player in nutrient cycling [18]. The potential effect of mangrove roots on sediment proteobacterial populations may influence several environmentally relevant processes in mangrove ecosystems. Root production in a tropical mangrove dominated by *R. mangle* can also be much higher than in inland forests; mangrove roots form a complex below-ground net with a growth of about 28 tons of dry biomass per hectare per year [19], [20]. The ability of such root systems to facilitate the growth of specific microbial guilds, may be essential for nutrient cycling and ecological resilience.

Our results showed that specific proteobacterial groups involved in the biogeochemical sulphur cycle were the most abundant bacterial guilds in the mangrove samples. The *Desulfobacterales* was the most abundant order detected in Trn, Nat and Bul samples. This order encompasses primarily sulfate-reducing bacteria (SRB) which are important players in the process of anoxic mineralization of organic matter and pollutants, such as anthropogenic hydrocarbons [21], [22]. *Chromatiales* was the second most abundant proteobacterial order and was detected in all mangrove samples (but not in nursery samples). This order is represented by anaerobic or microaerophilic microorganisms specialized in sulfur-anoxygenic photosynthesis and are able to oxidize hydrogen sulfide (H_2_S) to elemental sulphur [23]. *Campylobacterales* were also abundant and mainly detected in the mangrove samples (Trn, Nat and Bul) with a marked increased abundance in rhizosphere samples. Members of this order are sulfide-oxidizing denitrifying bacteria [24].

The ternary plots of dominant OTU's also showed increased abundance of the *Campylobacterales* belonging to the genus *Sulfurovum* in mangrove rhizosphere samples. This genus is known to be an important player in the process of sulfide-oxidation and denitrification in marine environments [24]–[26]. A previous study [27] also showed that *R. mangle* can oxidize the sediment rhizosphere and thereby contribute to the reduction of hydrogen sulfide in the sediment. However, no study has investigated the potential role of plant microbe interactions in the process of sulfide sediment detoxification in mangrove ecosystems. Our results reveal for the first time that *R. mangle* roots appear to be able to enhance the abundance of bacterial sulfide oxidizers which in turn may have further ecological implications for the process of sediment sulfide detoxification.

Curiously, the RDP and ternary plot analyses showed that *Rhizobiales* populations were more abundant in nursery and transplant samples than in native and bulk sediment samples. These results indicate that the nursery period was important for recruitment of nitrogen-fixing rhizobia. Such phenomena can favour the growth of mangrove replants in nitrogen-poor mangrove sediment. Mangrove rhizospheres (Trn and Nat) also showed a preferential enhancement of OTU's assigned to the *Methylococcaceae* family in comparison to bulk sediment samples, but in contrast to the *Rhizobiales*, members of this family were absent from nursery samples. Previous studies have shown that the *Methylococcaceae* family encompasses aerobic methanotrophs, which are key players in the methane flux from sediment (marine and fresh water) to the atmosphere [28]. Our results suggest an important ecological role of *R. mangle* roots in the selective enhancement of methanotrophic populations in mangroves. The chemical properties of the rhizosphere can have a strong influence on microbial activity and thus affect several processes of environmental relevance [29]. The effect of nursery conditions and roots on the diversity and abundance of methane consuming bacteria in the sediment surrounding the roots of mangrove plants has not been previously demonstrated. Such an effect can be important when considering the global destruction of mangrove habitat and large scale replanting approaches and merits further study.

Our analyses also suggest that mangrove roots are a preferred habitat for *Vibrio* populations. The *Vibrio* genus includes mainly aquatic bacteria, several of which are free-living and obligate endosymbionts. Previous studies on nitrogen-fixation in mangrove ecosystems have already identified a number of *Vibrio* species in the rhizosphere of mangroves [30]. However, none of these studies made comparative analyses of their relative abundance in mangrove rhizosphere (transplanted and native) versus bulk sediment samples.

Several dominant OTU's were only detected in nursery or transplanted samples, strengthening our observation that rhizobacterial populations acquired during nursery growth were introduced into the mangrove environment and remained abundant in the rhizosphere up to 202 dap. The ability of rhizo-competent bacteria to survive during the first months of transplantation is an important finding because this phase is the most critical for sapling survival [31]. Anything that can significantly augment the transplantation success of mangrove saplings will be of major importance to the conservation and restoration of this important ecosystem. We also hope that our study will function as a catalyst to stimulate long-term studies to understand how microbial communities change through time in mangrove environments including the impact of transplantation on community dynamics.

In conclusion, our results reveal a strong treatment effect and marked heterogeneity in OTU composition. An important finding from this study is the observation that rhizo-competent bacteria are able to colonize mangrove roots while the plants are still in the nursery and are able to survive in the mangrove rhizosphere for an extended period of time after transplantation. This is the first study to demonstrate such an effect and suggests that the initial conditions in which saplings are raised can have a pronounced and long-term effect on the root microbial community. This effect may help to explain the often highly variable success rate of reforestation projects since both plant growth promoters and plant pathogens may be introduced into the mangrove rehabilitation area. A more thorough understanding of how nursery conditions affect the microbial communities of transplants may yield new insights into the potential of this phenomenon for the restoration of degraded mangrove forests. The recent development of molecular techniques such as massive parallel pyrosequencing will greatly contribute to this task. Our results also contribute to elucidate the role of mangrove roots as a habitat for estuarine sediment bacteria.

## Materials and Methods

Initially a replanting approach was simulated in an urban mangrove located in Guanabara Bay (Rio de Janeiro, Brazil) (22°46′53″S/43°04′16″W). The sampling site characteristics have been described previously [32]. Mature propagules of the mangrove tree species *Rhizophora mangle* were collected from mangrove forests located in Guanabara Bay and planted in polyethylene bags containing a mixture of clay mineral and red yellow podzolic soil (1∶1). This mixture has been used successfully for almost a decade in mangrove replanting projects in Rio de Janeiro (Brazil), with plants supplied by José Luiz de Castro Ferreira (Association ‘Amigos do Manguezal de Jequiá’, Rio de Janeiro, Brazil). The plants were watered every day with fresh water and marine water (3 times each) during 75 days. The use of a soil mixture as substrate instead of mangrove sediment allows us to evaluate whether distinct initial growing conditions would have a long-term effect on the microbial communities of transplants. Before replanting, the saplings were carefully removed from the plastic bags to avoid damage to the root system; loose soil, i.e., not adhering to the roots, was discarded. Four replicate samples were made of (1) the roots of nursery plants before planting (Nur), (2) roots of transplanted saplings 202 dap (Trn), (3) roots of native (non-transplanted) saplings (Nat) and (4) bulk sediment in the replant area (each consisting of four cores ∼20 cm of top sediment with 4 cm diameter) (Bul). The transplanted plants appeared healthy and were approximately 50 cm in height. An effort was made to retrieve native saplings in a similar condition and growth stage to the transplanted saplings. Replicate samples of bulk sediment, native and transplanted saplings were made haphazardly over an area of 10 m^2^ and care was taken that replicates from a given treatment were not clustered together so as to avoid pseudo-replication. Each rhizosphere sample consisted of the total root system with tightly adhering sediment of each individual plant [16]. A spatula was used to remove the sediment that could be easily detached from the roots. Only sediment adhering to the plant root system was considered as the rhizosphere fraction. Each rhizosphere sample consisted of the total root system. Each sample was thoroughly mixed and microbial cells were detached from rhizosphere and bulk sediment samples (5 grams) as previously described [33]. The microbial pellet was obtained and total community DNA extraction was performed using a BIO-101 DNA extraction kit (Q Biogene) and mechanical lysis [33].

A barcoded pyrosequencing approach was used for characterization of bacterial communities. The V4 hyper-variable region of the bacterial 16S rRNA gene was PCR amplified for each sample (∼248 bp) using primers and tags described in the pyrosequencing pipeline of the Ribosomal Database Project (RDP) (Release 10, Update 20) (http://rdp.cme.msu.edu/). Pyrosequencing libraries were obtained using the 454 Genome Sequencer FLX platform (Roche Diagnostics Ltd, West Sussex, UK). Only sequences containing exact matches to primer sequences and barcode tags were used for further analyses. The primers were trimmed and sequences with reads below 150 bp or with ambiguous bases were discarded. The relative abundance of the most dominant bacterial groups in each treatment and the representative sequences of the most dominant OTU's (≥50 reads) were determined according to the Naive Bayesian rRNA Classifier (Version 1.0) of the RDP (Release 10, Update 20) with 50% as bootstrap cut-off. The results of this bootstrap value are close to the ones with 80% cut-off [33]. Sequences classified as plant organelles or not classified into the *Bacteria* domain were removed. After quality control, the sequencing effort yielded 5940, 10443, 6828 and 7428 reads for the treatments Nur, Trn, Nat and Bul, respectively.

The selected pyrosequencing reads were aligned online using the INFERNAL aligner algorithm [34]. Aligned sequences were assigned (97% identity) to OTU's (phylotype clusters) using the Complete Linkage Clustering application of the RDP pyrosequencing pipeline [35]. The complete linkage cluster file was then converted into a square matrix containing the presence and abundance of OTU's per sample using a self-written function in R (Supplementary Data S1). All 454 sequences generated in this study can be downloaded from the NCBI Short Read Archive, accession number: SRA023845.

The OTU richness rarefaction curve of each sample was computed using a self-written function in R (Supplementary Data S2). Dominance-diversity plots were generated based on the logarithmic species abundance against the rank order of species for each sample using the radfit() function in the vegan package [36]. Best fit lines representing the Zipf and Zipf-Madelbrot models were automatically generated. The Zipf model is a generalized linear model (‘glm’) with logarithmic link function whereas the Zipf-Mandelbrot adds one nonlinear parameter to the Zipf model. For an explanation of the ecological mechanisms behind the models see Wilson [37] although it should be noted that a good model fit does not necessarily imply a given mechanism. Variation in composition among treatments was assessed with Principal coordinates analysis (PCO), using the cmdscale() function in the R base package and wascores() function in vegan. Prior to the PCO, the raw data was log_10_ (*x*+1) transformed and used to produce a distance matrix based on the Hellinger distance with the decostand() function in vegan and dist() base R function. Variation in OTU composition among treatments was tested for significance using the adonis() function in vegan. The adonis() function is an analysis of variance with distance matrices using permutations that partitions distance matrices among sources of variation; in this case treatments. In the adonis() analysis, the Hellinger distance matrix of OTU composition was the response variable with treatment as independent variable. The number of permutations was set at 999; all other arguments used the default values set in the function. Variation in the relative abundance of dominant higher taxa was tested for significance with an analysis of deviance using the glm() function in R. Because the data was proportional, we first applied a glm with the family =  argument set as binomial. The ratio, however, of residual deviance to residual d.f. in the models substantially exceeded 1 so we set family =  to ‘quasibinomial’. In the ‘quasibinomial’ family the dispersion parameter is not fixed at one so that it can model over-dispersion.

Variation in the distribution of the most dominant taxa (≥50 reads) among treatments was assessed using ternary diagrams representing the percent abundance of dominant bacterial OTU's as determined by complete linkage cluster analysis of 16S rRNA gene sequences. The ternary diagrams were obtained using the ternaryplot() function of the vcd package in R.

## Supporting Information

Data S1 R self-written function for conversion of complete linkage cluster files (RDP pyrosequencing pipeline) into a square matrix containing the presence and abundance of OTU's per sample.
(0.02 MB PDF)

Data S2 R self-written function for construction of OTU richness rarefaction curves.
(0.02 MB PDF)
