# Supplementary material for: Taking Root: Enduring Effect of Rhizosphere Bacterial Colonization in Mangroves
Source: PLoS One. 2010 Nov 22;5(11):e14065. doi: 10.1371/journal.pone.0014065 (PMC2989908; doi:10.1371/journal.pone.0014065)
Supplement: Data S1 — R self-written function for conversion of complete linkage cluster files (RDP pyrosequencing pipeline) into a square matrix containing the presence and abundance of OTU's per sample. (0.02 MB PDF) [file pone.0014065.s001.pdf]

```r
# The 'Matrix.clust' function will convert output obtained from complete
linkage cluster application of the RDP pyrosequencing pipeline to a workable
square matrix format that can be then used within R to study phenomena
including species richness and community composition as outlined in the paper.


# NOTES

# The input file is a tab delimited text file that consists of two columns. The
first 'Number' is the number of individuals sampled per 'OTU'. The next column
'Cluster' gives the codes of each sequence assigned to a given OTU. Each
sequence is separated by a space. When imported into R using the read.table
function, this will produce a 999 x 2 data frame of two columns where the first
is a numeric column consisting of a single number per row and the second column
is a character column (i.e., class 'character') whereby each row contains a
variable length character string depending on the number of sequences assigned
to a given OTU. Each sequence is separated by a space. For those with little
previous experience using R: when preparing your own input file for importation
into R, make sure it follows this two column format using the appropriate
headers to avoid errors. The 'Labels' file contains the labels used to identify
sequences from different samples/treatments. The function will produce a column
for each label. Needless to say labels associated with samples will need to
have been included in the sequence names! Once the Matrix.clust function has
been pasted/piped into R, you can execute it as shown below. This will produce
a 999 x 16 matrix in R with columns identified by a sample 'label' and rows
representing OTU's as in the input file.

# In order to test the function (see below), you need to first
# I paste/pipe the function into R
# II import the data files
# III execute the function

# Daniel Cleary


# THE ACTUAL FUNCTION

"Matrix.clust"<-function(clust.vec,sites.codes) {

# clust.vec is a single vector containing the complete linkage cluster (RDP)
output. Individual sequences are separated by an empty space (blank) in the
example. If other symbols are used to separate sequences, then this can be
modified in the clust.list() internal function
# sites.codes is a single vector containing the four letter codes identifying
each sample site or treatment. You can use codes of different length by
altering the substr function in the internal function.

clust.char<-as.character(clust.vec)
clust.list<-strsplit(clust.char," ") # character separating sequences; in this
case an empty space

clust.max <- max(sapply(clust.list, length))
clust.matrix<-sapply(clust.list, function (x) c(x, rep(NA, clust.max -
length(x))))# make a square matrix with dimension clust.max
colnames.clust.matrix<-1:ncol(clust.matrix)
colnames(clust.matrix)<-colnames.clust.matrix

out.clust<-matrix(NA,length(clust.vec),length(sites.codes))


# Internal function to calculate species abundances for a single sample
spec.site<-function(clust.matrix.in,sites.codes.in) {
```

```
    SITE<-apply(clust.matrix.in,2,function (x) {ifelse(substr(x,1,
4)==sites.codes.in,1,0)})
    SITE[is.na(SITE)]<-0
    SITE.sum<-apply(SITE,2,sum)
    SITE.sum
              }

for(i in 1:length(sites.codes))   {

      out.clust[,i]<-spec.site(clust.matrix,sites.codes[i])
      colnames(out.clust)<-sites.codes
          }
return(out.clust)
}


# TEST

# IMPORT DATA

cluster.mangrove<-
read.table("http://sites.google.com/site/dfrcleary/resources/files/clust_mangrove_sample
 header=TRUE, as.is=TRUE)
otherwise you'll get an import error
Labels<-
read.table("http://sites.google.com/site/dfrcleary/resources/files/Labels_new.txt",
 header=TRUE, na.strings = "NA")

# EXECUTE FUNCTION

output.clust.matrix<-Matrix.clust(cluster.mangrove$Cluster,rownames(Labels))
colnames(output.clust.matrix)<-c("Trna","Trnb","Trnc","Trnd","Nura","Nurb",
"Nurc","Nurd","Nata","Natb","Natc","Natd","Bula","Bulb","Bulc","Buld")

# Replace old sample names used in sequence with new ones
```
