# Supplementary material for: Taking Root: Enduring Effect of Rhizosphere Bacterial Colonization in Mangroves
Source: PLoS One. 2010 Nov 22;5(11):e14065. doi: 10.1371/journal.pone.0014065 (PMC2989908; doi:10.1371/journal.pone.0014065)
Supplement: Data S2 — R self-written function for construction of OTU richness rarefaction curves. (0.02 MB PDF) [file pone.0014065.s002.pdf]

```
# THE 'rarefy.rich' FUNCTION

# The 'rarefy.rich' function using resampling to generate rarefaction curves
whereby points along the 'curve' represent estimated species richness for a
given sample size and given number of permutations. Confidence intervals and
standard deviations for these points are also given. The input matrix consists
of a table whereby columns represent samples (plots or time periods) and rows
species. The values in each cell represent the number of times a given species
was observed in that cell.

# This function is a quick way of generating rarefaction curves for multiple
samples at a range of sample sizes.

# bootsize is the 'bootstrap size', that is the number of times a sample is
drawn from the complete population of species. A reasonable value for bootsize
is between 200 and 1000.

# sampsize is the 'sample size', the number of individuals for which, species
richness is calculated. This can include a single number or a vector of numbers,
 which will produce values to visualise sampling (rarefaction) curves.

# group is a vector, which has the same length as the columns in rawdata and
identifies groups of samples

# col is a character vector, which has the same length as the columns in
rawdata that can be used to colour code the groups

# the output 'rarefac' is a 'long' table that includes 5 columns, namely, the
sample size, sample name, mean, standard deviation, and 95% confidence
intervals. These mean, standard deviation and 95% confidence interval values
are estimated by the bootstrap procedure. Each row represents the values for
the points along the rarefaction curve. The number of points is determined by
the sampsize vector.

# PLEASE REFERENCE WHEN USING THIS FUNCTION: Gomes NCM, Cleary DFR, Pinto FN,
Egas C, Almeida A, Cunha A, MendonÃ§a-Hagler, Smalla K. 2010. Taking root:
enduring effect of rhizosphere bacterial colonization in mangroves. PLoS ONE

# Daniel Cleary and Emiel van Loon, October 2010, Aveiro Portugal


# FUNCTION BEGINS HERE

"rarefy.rich" <- function(rawdata, bootsize=NULL, sampsize=NULL, group=NULL,
col=NULL){

if(is.null(bootsize)){bootsize=1000}

if(is.null(sampsize)){sampsize=10}

if(is.null(group)){group="NA"}

if(is.null(col)){col="black"}

rarefac = array(NA,dim=c(3,ncol(rawdata), length(sampsize)), dimnames =
list(c("Mean","SD","CI"), colnames(rawdata),NULL))


# internal function to calculate rarefied species richness and other indices
rarefirst<- function (rawdata.in, bootsize.in, sampsize.in) {apply(rawdata,2,
function(x) {
```

```
 ci_norm <-function(x){se<-sqrt(var(x)/length(x))
    tvalue<-qt(.975,length(x))
    tvalue*se}

 # creating the 'unfolded' vector representing the number of occurences of each
'species'
 rareout=matrix(NA,3,ncol(rawdata))

 nrunique = rep(NA,bootsize.in)

 unfolded = rep(NA,sum(x))


 p = 1

   for (i in 1:length(x)){


       if (x[i]>0){

       nrindiv = x[i]

       unfolded[p:(p+nrindiv-1)] = rep(i,nrindiv)

       p = p+nrindiv }}



 nrunfold=length(unfolded)

 sampsize.in=min(nrunfold,sampsize.in)

 # the actual sampling out of the unfolded vector, and repeating this
'bootsize' times
 for(j in 1:bootsize.in){


     nrunique[j] = length( unique( unfolded[sample(1:nrunfold,sampsize.in)] ) )
}


 rareout[]<-rbind(mean(nrunique),sd(nrunique),ci_norm(nrunique))
}
)
}

# Repeating the index calculation for all values of 'sampsize'
for(k in 1:length(sampsize)){
rarefac[,,k]<-rarefirst(rawdata,bootsize,sampsize[k])
}

rarefac.sd<-rarefac[2,,]
rarefac.ci<-rarefac[3,,]
rarefac.mean<-rarefac[1,,]
rarefac.mean.fin<-as.vector(ifelse(rarefac.sd==0,NA,rarefac.mean))
rarefac.sd.fin<-as.vector(ifelse(rarefac.sd==0,NA,rarefac.sd))
```

```
rarefac.ci.fin<-as.vector(ifelse(rarefac.sd==0,NA,rarefac.ci))

groups<-rep(group,length(sampsize))
cols<-rep(col,length(sampsize))
sites<-rep(colnames(rawdata),length(sampsize))
sample.size<-rep(sampsize,each=length(colnames(rawdata)))



rarefac<-data.frame(groups,sites,cols,sample.size,rarefac.mean.fin,
rarefac.sd.fin,rarefac.ci.fin)
colnames(rarefac)<-c("Groups","Sample","Colours","Sample_size","Mean","SD","CI")


return(rarefac)
}

# FUNCTION ENDS HERE


# TEST FUNCTION BEGINS HERE

# In order to test the function we'll use the 'output.clust.matrix' object
created previously with the 'Matrix.clust' function. Be careful to have
modified the colnames as indicated.


# CREATE VECTORS OF GROUP  NAMES AND COLOUR CODES FOR EACH GROUP

Group.rar<-c("Trn","Trn","Trn","Trn","Nur","Nur","Nur","Nur","Nat","Nat","Nat",
"Nat","Bul","Bul","Bul","Bul")
Col.rar<-c("Green","Green","Green","Green","Blue","Blue","Blue","Blue","Red",
"Red","Red","Red","Brown","Brown","Brown","Brown")

# EXECUTE FUNCTION

output.rarefaction.results<-rarefy.rich(output.clust.matrix,bootsize=999,
sampsize=seq(from=100,to=2000,by=100),group=Group.rar,col=Col.rar)


# PRODUCE FIGURE SHOWING RAREFACTION CRUVES

library(plotrix)

plotCI(output.rarefaction.results$Sample_size,output.rarefaction.results$Mean,
pch=21, pt.bg=(as.character(output.rarefaction.results$Colours)),xlab=
size",ylab="Rarefied richness", ui=output.rarefaction.results$Mean +
output.rarefaction.results$SD,li= output.rarefaction.results$Mean -
output.rarefaction.results$SD)
legend(1800,110,legend=unique(output.rarefaction.results$Groups),pch=21,
pt.bg=unique(as.character(output.rarefaction.results$Colours)), bty="n")
```
